# Linear time minimum segmentation enables scalable founder reconstruction

**DOI:** 10.1186/s13015-019-0147-6

**Published:** 2019-05-17

**Authors:** Tuukka Norri, Bastien Cazaux, Dmitry Kosolobov, Veli Mäkinen

**Affiliations:** 1 Department of Computer Science, University of Helsinki, Pietari Kalmin katu 5, 00014 Helsinki, Finland; 2 Ural Federal University, Mira 19, Yekaterinburg, 620002 Russia

**Keywords:** Pan-genome indexing, Founder reconstruction, Dynamic programming, Positional Burrows–Wheeler transform, Range minimum query

## Abstract

**Background:**

We study a preprocessing routine relevant in pan-genomic analyses: consider a set of aligned haplotype sequences of complete human chromosomes. Due to the enormous size of such data, one would like to represent this input set with a few *founder* sequences that retain as well as possible the contiguities of the original sequences. Such a smaller set gives a scalable way to exploit pan-genomic information in further analyses (e.g. read alignment and variant calling). Optimizing the founder set is an NP-hard problem, but there is a segmentation formulation that can be solved in polynomial time, defined as follows. Given a threshold *L* and a set ${\mathcal {R}} = \{R_1, \ldots , R_m\}$ of *m* strings (haplotype sequences), each having length *n*, the minimum segmentation problem for founder reconstruction is to partition [1, *n*] into set *P* of disjoint segments such that each segment $[a,b] \in P$ has length at least *L* and the number $d(a,b)=|\{R_i[a,b] :1\le i \le m\}|$ of distinct substrings at segment [*a*, *b*] is minimized over $[a,b] \in P$. The distinct substrings in the segments represent founder blocks that can be concatenated to form $\max \{ d(a,b) :[a,b] \in P \}$ founder sequences representing the original ${\mathcal {R}}$ such that crossovers happen only at segment boundaries.

**Results:**

We give an *O*(*mn*) time (i.e. linear time in the input size) algorithm to solve the minimum segmentation problem for founder reconstruction, improving over an earlier $O(mn^2)$.

**Conclusions:**

Our improvement enables to apply the formulation on an input of thousands of complete human chromosomes. We implemented the new algorithm and give experimental evidence on its practicality. The implementation is available in https://github.com/tsnorri/founder-sequences.

## Background

A key problem in *pan-genomics* is to develop a sufficiently small, efficiently queriable, but still descriptive representation of the variation common to the subject under study [1]. For example, when studying human population, one would like to take all publicly available variation datasets (e.g. [2–4]) into account. Many approaches encode the variation as a graph [5–10] and then one can encode the different haplotypes as paths in this graph [11]. An alternative has been proposed [12] based on a compressed indexing scheme for a multiple alignment of all the haplotypes [13–17]. In either approach, scalability is hampered by the encoding of all the haplotypes.

We suggest to look for a smaller set of representative haplotype sequences to make the above pan-genomic representations scalable.

Finding such set of representative haplotype sequences that retain the original contiguities as well as possible, is known as the *founder sequence reconstruction* problem [18]. In this problem, one seeks a set of *d* founders such that the original *m* haplotypes can be mapped with minimum amount of *crossovers* to the founders. Here a crossover means a position where one needs to jump from one founder to another to continue matching the content of the haplotype in question. Unfortunately, this problem is ${\textsf {NP}}$-hard even to approximate within a constant factor [19].

For founder reconstruction to be scalable to the pan-genomic setting, one would need an algorithm to be nearly linear to the input size. With this is mind, we study a relaxation of founder reconstruction that is known to be polynomial time solvable: Namely, when limiting all the crossovers to happen at the same locations, one obtains a *minimum segmentation* problem specific to founder reconstruction [18]. A dynamic programming algorithm solves this problem in $O(n^2m)$ time [18], where *m* is the number of haplotypes and *n* is the length of each of them.

In this paper, we improve the running time of solving the minimum segmentation problem of founder reconstruction to *O*(*mn*) (linear in the input size).

We also implement the new algorithm, as well as a further heuristic that aims to minimize crossovers over the segment boundaries (yielded by the optimal solution to the minimum segmentation problem). In our experiments, we show that the approach is practical on human genome scale setting. Namely, we apply the implementation on a multiple alignment representing 5009 haplotypes of human chromosome 6, and the result is 130 founder sequences with the average distance of two crossovers being 9624 bases. Preserving such long contiguities in just 2.5% of the original input space is promising for the accuracy and scalability of the short read alignment and variant calling motivating our study.

The main technique behind the improvement is the use of *positional Burrows–Wheeler transform* (pBWT) [20], and more specifically its extension to larger alphabets [21]. While the original dynamic programming solution uses *O*(*nm*) time to look for the best preceding segment boundary for each column of the input, we observe that at most *m* values in pBWT determine segment boundaries where the number of distinct founder substrings change. Minimums on the already computed dynamic programming values between each such interesting consecutive segment boundaries give the requested result. However, it turns out that we can maintain the minimums directly in pBWT internal structures (with some modifications) and have to store only the last *L* computed dynamic programming values, thus spending only $O(m + L)$ additional space, where *L* is the input threshold on the length of each segment. The segmentation is then reconstructed by standard backtracking approach in *O*(*n*) time using an array of length *n*.

Preliminary version of this work appeared in WABI 2018 [22].

## Methods

### Notation and problem statement

For a string $s = c_1 c_2 \cdots c_n$, denote by |*s*| its length *n*. We write *s*[*i*] for the letter $c_i$ of *s* and *s*[*i*, *j*] for the *substring* $c_i c_{i + 1} \ldots c_j$. An analogous notation is used for arrays. For any numbers *i* and *j*, the set of integers $\{x \in {\mathbb {Z}} :i \le x \le j\}$ (possibly empty) is denoted by [*i*, *j*].

The input for our problem is the set ${\mathcal {R}} = \{R_1,\ldots ,R_m\}$ of strings of length *n*, called *recombinants*. A set ${\mathcal {F}} = \{F_1,\ldots ,F_d\}$ of strings of length *n* is called a *founder set* of ${\mathcal {R}}$ if for each string $R_i \in {\mathcal {R}}$, there exists a partition $P_i$ of the segment [1, *n*] into disjoint subsegments such that, for each $[a,b] \in P_i$, the string $R_i[a,b]$ is equal to $F_j[a,b]$ for some $j \in [1,d]$. The partition $P_i$ together with the mapping of the segments $[a, b] \in P_i$ to substrings $F_j[a, b]$ is called a *parse of* $R_i$ *in terms of* ${\mathcal {F}}$, and a set of parses for all $R_i \in {\mathcal {R}}$ is called a *parse of* ${\mathcal {R}}$ *in terms of* ${\mathcal {F}}$. The integers *a* and $b + 1$, for $[a,b] \in P_i$, are called *crossover points*; thus, in particular, 1 and $n + 1$ are always crossover points.

It follows from the definition that, in practice, it makes sense to consider founder sets only for pre-aligned recombinants. Throughout the paper we implicitly assume that this is the case, although all our algorithms, clearly, work in the unaligned setting too but the produce results may hardly make any sense.

We consider the problem of finding a “good” founder set ${\mathcal {F}}$ and a “good” corresponding parse of ${\mathcal {R}}$ according to a reasonable measure of goodness. Ukkonen [18] pointed out that such measures may contradict each other: for instance, a minimum founder set obviously has size $d = \max _{j\in [1,n]} \vert \{R_1[j], \ldots , R_m[j]\} \vert$, but parses corresponding to such set may have unnaturally many crossover points; conversely, ${\mathcal {R}}$ is a founder set of itself and the only crossover points of its trivial parse are 1 and $n + 1$, but the size *m* of this founder set is in most cases unacceptably large. Following Ukkonen’s approach, we consider compromise parameterized solutions. The *minimum founder set problem* is, given a bound *L* and a set of recombinants ${\mathcal {R}}$, to find a smallest founder set ${\mathcal {F}}$ of ${\mathcal {R}}$ such that there exists a parse of ${\mathcal {R}}$ in terms of ${\mathcal {F}}$ in which the distance between any two crossover points is at least *L* (the crossover points may belong to parses of different recombinants, i.e., for $[a, b] \in P_i$ and $[a', b'] \in P_j$, where $P_i$ and $P_j$ are parses of $R_i$ and $R_j$, we have either $a = a'$ or $|a - a'| \ge L$).

It is convenient to reformulate the problem in terms of segmentations of ${\mathcal {R}}$. A *segment* of ${\mathcal {R}} = \{R_1,\ldots ,R_m\}$ is a set ${\mathcal {R}}[j,k] = \{R_i[j,k] :R_i \in {\mathcal {R}}\}$. A *segmentation* of ${\mathcal {R}}$ is a collection *S* of disjoint segments that covers the whole ${\mathcal {R}}$, i.e., for any distinct ${\mathcal {R}}[j,k]$ and ${\mathcal {R}}[j',k']$ from *S*, [*j*, *k*] and $[j',k']$ do not intersect and, for each $x \in [1,n]$, there is ${\mathcal {R}}[j,k]$ from *S* such that $x \in [j,k]$. The *minimum segmentation problem* [18] is, given a bound *L* and a set of recombinants ${\mathcal {R}}$, to find a segmentation *S* of ${\mathcal {R}}$ such that $\max \{\vert {\mathcal {R}}[j,k] \vert :{\mathcal {R}}[j,k] \in S\}$ is minimized and the length of each segment from *S* is at least *L*; in other words, the problem is to compute

1 $$\begin{aligned} \min \limits _{S\in S_L} \max \{\vert {\mathcal {R}}[j,k] \vert :{\mathcal {R}}[j,k] \in S \}, \end{aligned}$$

where $S_L$ is the set of all segmentations in which all segments have length at least *L*.

The minimum founder set problem and the minimum segmentation problem are connected: any segmentation *S* with segments of length at least *L* induces in an obvious way a founder set of size $\max \{ \vert {\mathcal {R}}[j,k] \vert :{\mathcal {R}}[j,k] \in S \}$ and a parse in which all crossover points are located at segment boundaries (and, hence, at distance at least *L* from each other); conversely, if ${\mathcal {F}}$ is a founder set of ${\mathcal {R}}$ and $\{j_1, \ldots , j_p\}$ is the sorted set of all crossover points in a parse of ${\mathcal {R}}$ such that $j_q - j_{q-1} \ge L$ for $q \in [2,p]$, then $S = \{{\mathcal {R}}[j_{q-1},j_q{-}1] :q \in [2,p]\}$ is a segmentation of ${\mathcal {R}}$ with segments of length at least *L* and $\max \{ \vert {\mathcal {R}}[j,k] \vert :{\mathcal {R}}[j,k] \in S \} \le |{\mathcal {F}}|$.

Our main result is an algorithm that solves the minimum segmentation problem in *O*(*mn*) time (linear in the input size). The solution normally does not uniquely define a founder set of ${\mathcal {R}}$: for instance, if the built segmentation of ${\mathcal {R}} = \{baaaa, baaab, babab\}$ is $S = \{{\mathcal {R}}[1,1], {\mathcal {R}}[2,3], {\mathcal {R}}[4,5]\}$, then the possible founder sets induced by *S* are ${\mathcal {F}}_1 = \{baaab, babaa\}$ and ${\mathcal {F}}_2 = \{baaaa, babab\}$. In other words, to construct a founder set, one concatenates fragments of recombinants corresponding to the found segments in a certain order. We return to this ordering problem in the section describing experiments and now focus on the details of the segmentation problem.

Hereafter, we assume that the input alphabet $\Sigma$ is the set $[0,|\Sigma |{-}1]$ of size *O*(*m*), which is a natural assumption considering that the typical alphabet size is 4 in our problem. It is sometimes convenient to view the set ${\mathcal {R}} = \{R_1, \ldots , R_m\}$ as a matrix with *m* rows and *n* columns. We say that an algorithm processing the recombinants ${\mathcal {R}}$ is *streaming* if it reads the input from left to right “columnwise”, for each *k* from 1 to *n*, and outputs an answer for each set of recombinants $\{R_1[1,k], \ldots , R_m[1,k]\}$ immediately after reading the “column” $\{R_1[k], \ldots , R_m[k]\}$. The main result of the paper is the following theorem.

#### **Theorem 1**

*Given a bound* *L* *and recombinants* ${\mathcal {R}} = \{R_1, \ldots , R_m\}$, *each having length* *n*, *there is an algorithm that computes* (1) *in a streaming fashion in* *O*(*mn*) *time and* $O(m + L)$ *space. Using an additional array of length* *n*, *one can also find in* *O*(*n*) *time a segmentation on which* (1) *is attained, thus solving the minimum segmentation problem.*

### Minimum segmentation problem

Given a bound *L* and a set of recombinants ${\mathcal {R}} = \{R_1, \ldots , R_m\}$ each of which has length *n*, Ukkonen [18] proposed a dynamic programming algorithm that solves the minimum segmentation problem in $O(m n^2)$ time based on the following recurrence relation:

2 $$\begin{aligned} M(k) = {\left\{ \begin{array}{ll} +\infty & \text { if }k < L,\\ \vert {\mathcal {R}}[1,k] \vert & \text { if }L \le k < 2L, \\ \min \limits _{0 \le j \le k-L} \max \{M(j),\vert {\mathcal {R}}[j+1,k] \vert \} & \text { if } k \ge 2L. \end{array}\right. } \end{aligned}$$

It is obvious that *M*(*n*) is equal to the solution (1); the segmentation itself can be reconstructed by “backtracking” in a standard way [18]. We build on the same approach.

For a given $k \in [1,n]$, denote by $j_{k,1}, \ldots , j_{k,r_k}$ the sequence of all positions $j \in [1, k - L]$ in which the value of $|{\mathcal {R}}[j,k]|$ changes, i.e., $1 \le j_{k,1}< \cdots < j_{k,r_k} \le k - L$ and $|{\mathcal {R}}[j_{k,h},k]| \ne |{\mathcal {R}}[j_{k,h}{+}1,k]|$ for $h \in [1,r_k]$. We complement this sequence with $j_{k,0} = 0$ and $j_{k,r_k+1} = k - L + 1$, so that $j_{k,0}, \ldots , j_{k,r_k+1}$ can be interpreted as a splitting of the range $[0,k - L]$ into segments in which the value $\vert {\mathcal {R}}[j+1,k] \vert$ stays the same: namely, for $h \in [0,r_k]$, one has $\vert {\mathcal {R}}[j + 1,k] \vert = \vert {\mathcal {R}}[j_{k,h+1},k] \vert$ provided $j_{k,h} \le j < j_{k,h+1}$. Hence, $\min \nolimits _{j_{k,h} \le j< j_{k,h+1}} \max \{M(j), \vert {\mathcal {R}}[j + 1,k] \vert \} = \max \{\vert {\mathcal {R}}[j_{k,h+1},k] \vert , \min \nolimits _{j_{k,h} \le j < j_{k,h+1}} M(j)\}$ and, therefore, (2) can be rewritten as follows:

3 $$\begin{aligned} M(k) = {\left\{ \begin{array}{ll} +\infty & \text { if }k< L,\\ \vert {\mathcal {R}}[1,k] \vert & \text { if } L \le k< 2L,\\ \min \limits _{0 \le h \le r_k} \max \{\vert {\mathcal {R}}[j_{k,h+1},k] \vert , \min \limits _{j_{k,h} \le j < j_{k,h+1}} M(j)\} & \text { if } k \ge 2L. \end{array}\right. } \end{aligned}$$

Our crucial observation is that, for $k \in [1,n]$ and $j \in [1,k]$, one has $\vert {\mathcal {R}}[j + 1, k] \vert \le \vert {\mathcal {R}}[j, k] \vert \le m$. Therefore, $m \ge \vert {\mathcal {R}}[j_{k,1},k] \vert> \cdots > \vert {\mathcal {R}}[j_{k,r_k+1},k] \vert \ge 1$ and $r_k < m$. Hence, *M*(*k*) can be computed in *O*(*m*) time using (3), provided one has the following components:

i. the sorted sequence $j_{k,1}, \ldots , j_{k,r_k}$
ii. the numbers $\vert {\mathcal {R}}[j_{k,h+1},k] \vert$, for $h \in [0,r_k]$
iii. the values $\min \{ M(j) :j_{k,h} \le j < j_{k,h+1} \}$, for $h \in [0,r_k].$

In the remaining part of the section, we describe a streaming algorithm that reads the strings $\{R_1, \ldots , R_m\}$ “columnwise” from left to right and computes the components (i), (ii), and (iii) immediately after reading each “column” $\{R_1[k], \ldots , R_m[k]\}$, for $k \in [1,n]$, and all in *O*(*mn*) total time and $O(m + L)$ space.

To reconstruct a segmentation corresponding to the found solution *M*(*n*), we build along with the values *M*(*k*) an array of size *n* whose *k*th element, for each $k \in [1,n]$, stores 0 if $M(k) = \vert {\mathcal {R}}[1,k] \vert$, and stores a number $j \in [1,k{-}L]$ such that $M(k) = \max \{M(j), \vert {\mathcal {R}}[j{+}1,k] \vert \}$ otherwise; then, the segmentation can be reconstructed from the array in an obvious way in *O*(*n*) time. In order to maintain the array, our algorithm computes, for each $k \in [1,n]$, along with the values $\min \{ M(j) :j_{k,h} \le j < j_{k,h+1} \}$, for $h \in [0,r_k]$, positions *j* on which these minima are attained (see below). Further details are straightforward and, thence, omitted.

#### Positional Burrows–Wheeler transform

Let us fix $k \in [1,n]$. Throughout this subsection, the string $R_i[k] R_i[k-1] \cdots R_i[1]$, which is the reversal of $R_i[1,k]$, is denoted by $R'_{i,k}$, for $i \in [1,m]$. Given a set of recombinants ${\mathcal {R}} = \{R_1, \ldots , R_m\}$ each of which has length *n*, a *positional Burrows–Wheeler transform (pBWT)*, as defined by Durbin [20], is a pair of integer arrays $a_k[1,m]$ and $d_k[1,m]$ such that:

1. $a_k[1,m]$ is a permutation of [1, *m*] such that $R'_{a_k[1],k} \le \cdots \le R'_{a_k[m],k}$ lexicographically;
2. $d_k[i]$, for $i \in [1,m]$, is an integer such that $R_{a_k[i]}[d_k[i], k]$ is the longest common suffix of $R_{a_k[i]}[1,k]$ and $R_{a_k[i-1]}[1,k]$, and $d_k[i] = k + 1$ if either this suffix is empty or $i = 1$.

##### *Example 1*

**Fig. 1:**
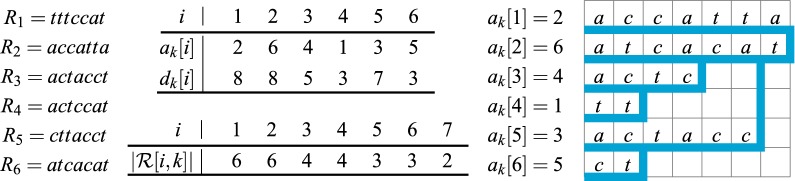
The pBWT for a set of recombinants ${\mathcal {R}} = \{R_1, \ldots , R_6\}$ with $k = 7$ and the corresponding trie containing the reversed strings $R_1[1,k], \ldots , R_6[1,k]$ in lexicographic order

Consider the following example, where $m = 6$, $k = 7$, and $\Sigma = \{a,c,t\}$. It is easy to see that the pBWT implicitly encodes the trie depicted in the right part of Fig. 1, and such interpretation drives the intuition behind this structure: The trie represents the *reversed* sequences $R_1[1,k], \ldots , R_6[1,k]$ (i.e., read from right to left) in lexicographic order. Leaves (values $a_k$) store the corresponding input indices. The branches correspond to values $d_k$ (the distance from the root subtracted from $k+1$). Our main algorithm in this paper makes implicitly a sweep-line over the trie stopping at the branching positions.

Durbin [20] showed that $a_k$ and $d_k$ can be computed from $a_{k-1}$ and $d_{k-1}$ in *O*(*m*) time on the binary alphabet. Mäkinen and Norri [21] further generalized the construction for integer alphabets of size *O*(*m*), as in our case. For the sake of completeness, we describe in this subsection the generalized solution [21] (see Algorithm 1), which serves then as a basis for our main algorithm. We also present a modification of this solution (see Algorithm 2), which, albeit seems to be slightly inferior in theory (we could prove only $O(m\log |\Sigma |)$ time upper bound), showed better performance in practice and thus, as we believe, is interesting by itself. 
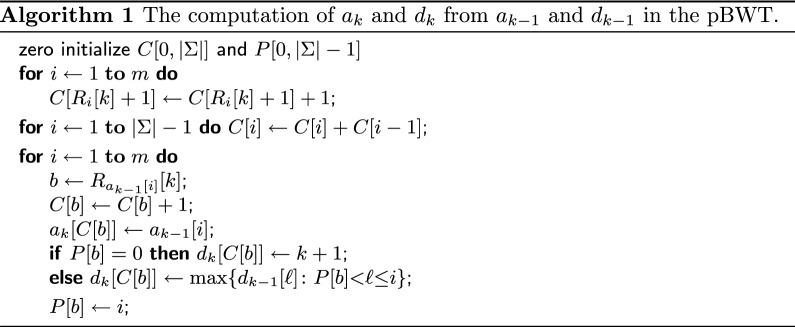
 
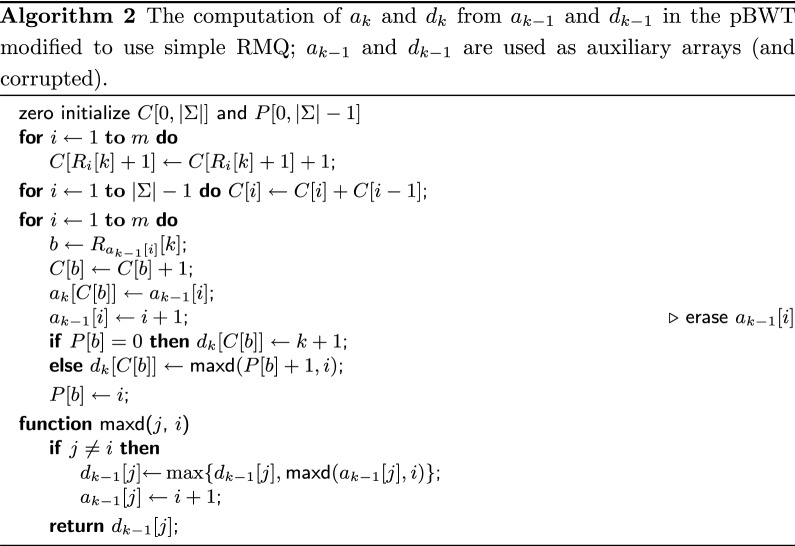


##### **Lemma 2**

*The arrays* $a_k[1,m]$ *and* $d_k[1,m]$ *can be computed from* $a_{k-1}[1,m]$ *and* $d_{k-1}[1,m]$ *in* *O*(*m*) *time, assuming the input alphabet is* $[0, |\Sigma |{-}1]$ *with* $|\Sigma |=O(m)$.

##### *Proof*

Given $a_{k-1}$ and $d_{k-1}$, we are to show that Algorithm 1 correctly computes $a_k$ and $d_k$. Since, for any $i, j \in [1,m]$, we have $R'_{i,k} \le R'_{j,k}$ iff either $R_i[k] < R_j[k]$, or $R_i[k] = R_j[k]$ and $R'_{i,k-1} \le R'_{j,k-1}$ lexicographically, it is easy to see that the array $a_k$ can be deduced from $a_{k-1}$ by radix sorting the sequence of pairs $\{ (R_{a_{k-1}[i]}[k], R'_{a_{k-1}[i],k-1}) \}_{i=1}^m$. Further, since, by definition of $a_{k-1}$, the second components of the pairs are already in a sorted order, it remains to sort the first components by the counting sort. Accordingly, in Algorithm 1, the first loop counts occurrences of letters in the sequence $\{R_i[k]\}_{i=1}^m$ using an auxiliary array $C[0,|\Sigma |]$; as is standard in the counting sort, the second loop modifies the array *C* so that, for each letter $b \in [0,|\Sigma |{-}1]$, $C[b] + 1$ is the first index of the “bucket” that will contain all $a_{k-1}[i]$ such that $R_{a_{k-1}[i]}[k] = b$; finally, the third loop fills the buckets incrementing the indices $C[b] \leftarrow C[b] + 1$, for $b = R_{a_{k-1}[i]}[k]$, and performing the assignments $a_k[C[b]] \leftarrow a_{k-1}[i]$, for $i = 1,\ldots ,m$. Thus, the array $a_k$ is computed correctly. All is done in $O(m + |\Sigma |)$ time, which is *O*(*m*) since the input alphabet is $[0,|\Sigma |{-}1]$ and $|\Sigma | = O(m)$.

The last three lines of the algorithm are responsible for computing $d_k$. Denote the length of the *longest common prefix* of any strings $s_1$ and $s_2$ by $\mathtt {LCP} (s_1, s_2)$. The computation of $d_k$ relies on the following well-known fact: given a sequence of strings $s_1, \ldots , s_r$ such that $s_1 \le \cdots \le s_r$ lexicographically, one has $\mathtt {LCP} (s_1, s_r) = \min \{\mathtt {LCP} (s_{i-1}, s_{i}) :1 < i \le r\}$. Suppose that the last loop of the algorithm, which iterates through all *i* from 1 to *m*, assigns $a_k[i'] \leftarrow a_{k-1}[i]$, for a given $i \in [1,m]$ and some $i' = C[b]$. Let *j* be the maximum integer such that $j < i$ and $R_{a_{k-1}[j]}[k] = R_{a_{k-1}[i]}[k]$ (if any). The definition of $a_k$ implies that $a_k[i' - 1] = a_{k-1}[j]$ if such *j* exists. Hence, $\mathtt {LCP} (R'_{a_k[i' - 1],k}, R'_{a_k[i'],k}) = 1 + \min \{\mathtt {LCP} (R'_{a_{k-1}[\ell - 1], k-1}, R'_{a_{k-1}[\ell ], k-1}) :j{<}\ell {\le }i\}$ if such number *j* does exist, and $\mathtt {LCP} (R'_{a_k[i' - 1],k}, R'_{a_k[i'],k}) = 0$ otherwise. Therefore, since $d_k[i']$ equals $k + 1 - \mathtt {LCP} (R'_{a_k[i'],k}, R'_{a_k[i'-1],k})$, we have either $d_k[i'] = \max \{d_{k-1}[\ell ] :j < \ell \le i\}$ or $d_k[i'] = k + 1$ according to whether the required *j* exists. To find *j*, we simply maintain an auxiliary array $P[0, |\Sigma |{-}1]$ such that on the *i*th loop iteration, for any letter $b \in [0,|\Sigma |{-}1]$, *P*[*b*] stores the position of the last seen *b* in the sequence $R_{a_{k-1}[1]}[k], R_{a_{k-1}[2]}[k], \ldots , R_{a_{k-1}[i-1]}[k]$, or $P[b] = 0$ if *b* occurs for the first time. Thus, $d_k$ is computed correctly.

In order to calculate the maximums $\max \{d_{k-1}[\ell ] :P[b] \le \ell \le i\}$ in *O*(1) time, we build a *range maximum query* (RMQ) data structure on the array $d_{k-1}[1,m]$ in *O*(*m*) time [23]. Therefore, the running time of Algorithm 1 is *O*(*m*). $\square$

In practice the bottleneck of the algorithm is the RMQ data structure, which, although answers queries in *O*(1) time, has a sensible constant under the big-O in the construction time. We could naively compute the maximums by scanning the ranges $d_{k-1}[P[b]{+}1,i]$ from left to right but such algorithm works in quadratic time since same ranges of $d_{k-1}$ might be processed many times in the worst case. Our key idea is to store the work done by a simple scanning algorithm to reuse it in future queries. We store this information right in the arrays $a_{k-1}$ and $d_{k-1}$ rewriting them; in particular, since $a_{k-1}$ is accessed sequentially from left to right in the last loop, the range $a_{k-1}[1,i]$ is free to use after the *i*th iteration.

More precisely, after the *i*th iteration of the last loop, the subarrays $a_{k-1}[1,i]$ and $d_{k-1}[1,i]$ are modified so that the following invariant holds: for any $j \in [1,i]$, $j < a_{k-1}[j] \le i + 1$ and $d_{k-1}[j] = \max \{d'_{k-1}[\ell ] :j \le \ell < a_{k-1}[j]\}$, where $d'_{k-1}$ denotes the original array $d_{k-1}$ before modifications; note that the invariant holds if one simply puts $a_{k-1}[j] = j + 1$ without altering $d_{k-1}[j]$. Then, to compute $\max \{d'_{k-1}[\ell ] :j\le \ell \le i\}$, we do not have to scan all elements but can “jump” through the chain $j, a_{k-1}[j], a_{k-1}[a_{k-1}[j]], \ldots , i$ and use maximums precomputed in $d_{k-1}[j], d_{k-1}[a_{k-1}[j]], d_{k-1}[a_{k-1}[a_{k-1}[j]]], \ldots , d_{k-1}[i]$; after this, we redirect the “jump pointers” in $a_{k-1}$ to $i + 1$ and update the maximums in $d_{k-1}$ accordingly. This idea is implemented in Algorithm 2. Notice the new line $a_{k-1}[i] \leftarrow i + 1$ in the main loop (it is commented), which erases $a_{k-1}[i]$ and makes it a part of the “jump table”. The correctness of the algorithm is clear. But it is not immediate even that the algorithm works in $O(m\log m)$ time. The next lemma states that the bound is actually even better, $O(m\log |\Sigma |)$.

##### **Lemma 3**

*Algorithm* 2 *computes the arrays* $a_k[1,m]$ *and* $d_k[1,m]$ *from* $a_{k-1}[1,m]$ *and* $d_{k-1}[1,m]$ *in* $O(m\log |\Sigma |)$ *time, assuming the input alphabet is* $[0, |\Sigma |{-}1]$ *with* $|\Sigma |=O(m)$.

##### *Proof*

Fix $i \in [1,m]$. The *i*th iteration of the last loop in the algorithm computes the maximum in a range $d'_{k-1}[i',i]$, where $d'_{k-1}$ is the original array $d_{k-1}$ before modifications and $i' = P[b] + 1$ for some *b* and *P*. Let $\ell _i = i - i'$. Denote ${\tilde{\ell }} = \frac{1}{m} \sum _{i=1}^m \ell _i$, the “average query length”. We are to prove that the running time of the algorithm is $O(m\log {\tilde{\ell }})$, which implies the result since $m{\tilde{\ell }} = \sum _{i=1}^m \ell _i$ and $\sum _{i=1}^m \ell _i \le |\Sigma | m$. The latter inequality follows from the fact that the query ranges correponding to the same symbol are non-overlapping.

**Fig. 2:**
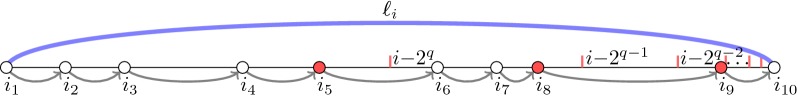
RMQ query on a range $[i - \ell _i, i]$; scaling touches are red

We say that a position *j* is *touched* if the function $\mathsf {maxd}$ is called with its first argument equal to *j*. Since for each *i* the first call to $\mathsf {maxd}$ is with different *j*, it suffices to prove that the total number of touches is $O(m\log {\tilde{\ell }})$. While processing the query $\mathsf {maxd}(i{-}\ell _i, i)$, we may have touched many positions. Denote the sequence of all such position, for the given *i*, by $i_1, \ldots , i_r$; in other words, at the time of the query $\mathsf {maxd}(i{-}\ell _i, i)$, we have $i_1 = i - \ell _i$, $i_j = a_{k-1}[i_{j-1}]$ for $j \in [2,r]$, $i_r = i$, and hence $i_1< \cdots < i_r$. We say that, for $j \in [1,r{-}1]$, the touch of $i_j$ in the query $\mathsf {maxd}(i{-}\ell _i, i)$ is *scaling* if there exists an integer *q* such that $i - i_j > 2^q$ and $i - i_{j+1} \le 2^q$ (see Fig. 2). We count separately the total number of scaling and non-scaling touches in all *i*.

For position *j*, denote by *p*(*j*) the number of non-scaling touches of *j*. We are to prove that $P = \sum _{j=1}^m p(j) \le 2 m \log {\tilde{\ell }}$. Let $q_h(j)$ denote the value of $a_{k-1}[j] - j$ in the *h*th non-scaling touch of *j*, for $h \in [1,p(j)]$. Suppose that this *h*th touch happens during the processing of a query $\mathsf {maxd}(i - \ell _i, i)$. By the definition, $j + q_h(j)$ follows *j* in the sequence of touched positions. Since the touch of *j* is non-scaling, we have $i-j> i-a_{k-1}[j]= i-j-q_h(j)> 2^q$, where *q* is the largest integer such that $i - j > 2^q$. Since $i-j\le 2^{q+1}$, there holds $q_h(j) < 2^q$. Since $\mathsf {maxd}(i - \ell _i, i)$ assigns $a_{k-1}[j] \leftarrow i + 1$, we have $a_{k-1}[j] - j> i - j > 2^q$ after the query. In other words, we had $a_{k-1}[j] - j = q_h(j) < 2^q$ before the query and have $a_{k-1}[j] - j > 2^q$ after. This immediately implies that $q_h(j) \ge 2^{h-1}$, for $h \in [1,p(j)]$, and, therefore, every position can be touched in the non-scaling way at most $O(\log m)$ times, implying $P = O(m\log m)$. But we can deduce a stronger bound. Since the sum of all values $a_{k-1}[j]-j$ for all positions *j* touched in a query $\mathsf {maxd}(i - \ell _i, i)$ is equal to $\ell _i$, we can bound the total sum of values $q_h(j)$ by $\sum _{j = 1}^m \sum _{h = 1}^{p(j)} q_h(j) \le \sum _{i=1}^m \ell _i = m{\tilde{\ell }}$. On the other hand, we have $\sum _{j = 1}^m \sum _{h = 1}^{p(j)} q_h(j) \ge \sum _{j = 1}^m \sum _{h = 1}^{p(j)} 2^{h-1} = \sum _{j=1}^m 2^{p(j)} - m$. The well-known property of the convexity of the exponent is that the sum $\sum _{j=1}^m 2^{p(j)}$ is minimized whenever all *p*(*j*) are equal, i.e., $\sum _{j=1}^m 2^{p(j)} \ge \sum _{j=1}^m 2^{P / m}$. Hence, once $P > 2 m \log {\tilde{\ell }}$, we obtain $\sum _{j = 1}^m \sum _{h = 1}^{p(j)} q_h(j) \ge \sum _{j = 1}^m 2^{P / m} - m > m{\tilde{\ell }}^2 - m$, which is larger than $m{\tilde{\ell }}$ for ${\tilde{\ell }} \ge 2$ (for the case ${\tilde{\ell }} < 2$ the claim follows directly), contradicting $\sum _{j = 1}^m \sum _{h = 1}^{p(j)} q_h(j) \le m{\tilde{\ell }}$. Thus, $P = \sum _{j=1}^m p(j) \le 2 m \log {\tilde{\ell }}$.

It remains to consider scaling touches. The definition implies that each query $\mathsf {maxd}(i{-}\ell _i, i)$ performs at most $\log \ell _i$ scaling touches. Thus, it suffices to upperbound $\sum _{i=1}^m \log \ell _i$. Since the function $\log$ is concave, the sum $\sum _{i=1}^m \log \ell _i$ is maximized whenever all $\ell _i$ are equal, i.e., $\sum _{i=1}^m \log \ell _i \le \sum _{i=1}^m \log (\frac{1}{m} \sum _{j=1}^m \ell _j) = m\log {\tilde{\ell }}$, hence the result follows. $\square$

#### Modification of the pBWT

We are to modify the basic pBWT construction algorithm in order to compute the sequence $j_{k,1}, \ldots , j_{k,r_k}$ of all positions $j \in [1,k-L]$ in which $\vert {\mathcal {R}}[j,k] \vert \ne \vert {\mathcal {R}}[j + 1,k] \vert$, and to calculate the numbers $\vert {\mathcal {R}}[j_{k,h+1},k] \vert$ and $\min \{ M(j) :j_{k,h} \le j < j_{k,h+1} \}$, for $h \in [0,r_k]$ (assuming $j_{k,0} = 0$ and $j_{k,r_k+1} = k - L + 1$); see the beginning of the section. As it follows from (3), these numbers are sufficient to calculate *M*(*k*), as defined in (2) and (3), in *O*(*m*) time. The following lemma reveals relations between the sequence $j_{k,1}, \ldots , j_{k,r_k}$ and the array $d_k$.

##### **Lemma 4**

*Consider recombinants* ${\mathcal {R}} = \{R_1, \ldots , R_m\}$, *each having length* *n*. *For* $k \in [1,n]$ *and* $j \in [1,k - 1]$, *one has* $\vert {\mathcal {R}}[j,k] \vert \ne \vert {\mathcal {R}}[j + 1,k] \vert$ *iff* $j = d_k[i] - 1$ *for some* $i \in [1,m]$.

##### *Proof*

Suppose that $\vert {\mathcal {R}}[j,k] \vert \ne \vert {\mathcal {R}}[j + 1,k] \vert$. It is easy to see that $\vert {\mathcal {R}}[j,k] \vert > \vert {\mathcal {R}}[j + 1,k] \vert$, which implies that there are two indices *h* and $h'$ such that $R_h[j + 1, k] = R_{h'}[j + 1, k]$ and $R_h[j] \ne R_{h'}[j]$. Denote by $a_k^{-1}[h]$ the number *x* such that $a_k[x] = h$. Without loss of generality, assume that $a_k^{-1}[h] < a_k^{-1}[h']$. Then, there exists $i \in [a_k^{-1}[h] + 1, a_k^{-1}[h']]$ such that $R_{a_k[i - 1]}[j + 1, k] = R_{a_k[i]}[j + 1, k]$ and $R_{a_k[i - 1]}[j] \ne R_{a_k[i]}[j]$. Hence, $d_k[i] = j + 1$.

Suppose now that $j \in [1, k - 1]$ and $j = d_k[i] - 1$, for some $i \in [1,m]$. Since $j < k$ and $d_k[1] = k + 1$, we have $i > 1$. Then, by definition of $d_k$, $R_{a_k[i-1]}[j + 1, k] = R_{a_k[i]}[j + 1, k]$ and $R_{a_k[i-1]}[j] \ne R_{a_k[i]}[j]$, i.e., $R_{a_k[i]}[j + 1, k]$ can be “extended” to the left in two different ways, thus producing two distinct strings in the set ${\mathcal {R}}[j, k]$. Therefore, $\vert {\mathcal {R}}[j, k] \vert > \vert {\mathcal {R}}[j + 1, k] \vert$. $\square$

Denote by *r* the number of distinct integers in the array $d_k$. Clearly, *r* may vary from 1 to *m*. For integer $\ell$, define $M'(\ell ) = M(\ell )$ if $1 \le \ell \le k - L$, and $M'(\ell ) = +\infty$ otherwise ($M'$ is introduced for purely technical reasons). Our modified algorithm does not store $d_k$ but stores the following four arrays (but we still often refer to $d_k$ for the sake of analysis):

- $s_k[1,r]$ contains all distinct elements from $d_k[1,m]$ in the increasing sorted order;
- $e_k[1,m]$: for $j \in [1,m]$, $e_k[j]$ is equal to the unique index such that $s_k[e_k[j]] = d_k[j]$;
- $t_k[1,r]$: for $j \in [1,r]$, $t_k[j]$ is equal to the number of times $s_k[j]$ occurs in $d_k[1,m]$;
- $$u_k[1,r]$$: for $$j \in [1,r]$$, $$u_k[j] = \min \{M'(\ell ) :s_k[j{-}1]{-}1 \le \ell < s_k[j]{-}1\}$$, assuming $$s_k[0] = 1$$.

The arrays $s_k$ and $e_k$ together emulate $d_k$. The array $t_k$ will be used to calculate some numbers $\vert {\mathcal {R}}[j, k] \vert$ required to compute *M*(*k*).

##### *Example 2*

In Example 1, where $m = 6$, $k = 7$, and $\Sigma = \{a,c,t\}$, we have $r = 4$, $s_k = [3, 5, 7, 8]$, $t_k = [2, 1, 1, 2]$, $e_k = [4, 4, 2, 1, 3, 1]$. It is easy to see that the array $s_k$ marks positions of the branching nodes in the trie from Fig. 1 in the increasing order (in the special case $s_k[1] = 1$, $s_k[1]$ does not mark any such node). Suppose that $L = 3$, so that $k - L = 4$. Then, $u_k[1] = M(1)$, $u_k[2] = \min \{M(2), M(3)\}$, $u_k[3] = \min \{M(4), M'(5)\} = M(4)$ since $M'(5) = +\infty$, and $u_k[4] = M'(6) = +\infty$. The use of $u_k$ is discussed in the sequel.

For convenience, let us recall Eq. (3) defined in the beginning of this section:

3 revisited $$\begin{aligned} M(k) = {\left\{ \begin{array}{ll} +\infty & \text { if }k< L,\\ \vert {\mathcal {R}}[1,k] \vert & \text { if } L \le k< 2L,\\ \min \limits _{0 \le h \le r_k} \max \{\vert {\mathcal {R}}[j_{k,h+1},k] \vert , \min \limits _{j_{k,h} \le j < j_{k,h+1}} M(j)\} & \text { if } k \ge 2L, \end{array}\right. } \end{aligned}$$

where $j_{k,0} = 0$, $j_{k,r_k + 1} = k - L + 1$, and $j_{k,1}, \ldots , j_{k,r_k}$ is the increasing sequence of all positions $j \in [1,k-L]$ in which $\vert {\mathcal {R}}[j,k] \vert \ne \vert {\mathcal {R}}[j + 1,k] \vert$. In order to compute *M*(*k*), one has to find the minima $\min \nolimits _{j_{k,h} \le j < j_{k,h+1}} M(j)$ and calculate $\vert {\mathcal {R}}[j_{k,h+1},k] \vert$. As it follows from Lemma 4 and the definition of $s_k$, all positions $j \in [1, k - 1]$ in which $\vert {\mathcal {R}}[j,k] \vert \ne \vert {\mathcal {R}}[j + 1,k] \vert$ are represented by the numbers $s_k[i] - 1$ such that $1 < s_k[i] \le k$ (in the increasing order); hence, the sequence $j_{k,1}, \ldots , j_{k,r_k}$ corresponds to either $s_k[1] - 1, \ldots , s_k[r_k] - 1$ or $s_k[2] - 1, \ldots , s_k[r_k + 1] - 1$, depending on whether $s_k[1] \ne 1$. Then, the minima $\min \nolimits _{j_{k,h} \le j < j_{k,h+1}} M(j)$ are stored in the corresponding elements of $u_k$ (assuming $s_k[0] = 1$): $u_k[i] = \min \{M'(\ell ) :s_k[i{-}1]{-}1 \le \ell< s_k[i]{-}1\} = \min \{M(\ell ) :s_k[i{-}1]{-}1 \le \ell< \min \{s_k[i]{-}1, k - L + 1\}\} = \min \nolimits _{j_{k,h} \le j < j_{k,h+1}} M(j)$, provided $s_k[i - 1] - 1 = j_{k,h}$. It is clear that $u_k[i] \ne +\infty$ only if the segment $[s_k[i - 1] - 1, s_k[i] - 2]$ intersects the range $[1, k - L]$ and, thus, corresponds to a segment $[j_{k,h}, j_{k,h+1} - 1]$, for $h \in [0,r_k]$. Therefore, since $M'(\ell ) = +\infty$ for $\ell < 1$ and $\ell > k - L$ and, thus, such values $M'(\ell )$ do not affect, in a sense, the minima stored in $u_k$, one can rewrite (3) as follows:

4 $$\begin{aligned} M(k) = {\left\{ \begin{array}{ll} +\infty & \text { if }k< L,\\ \vert {\mathcal {R}}[1,k] \vert & \text { if } L \le k < 2L,\\ \min \limits _{1 \le j \le |u_k|} \max \{\vert {\mathcal {R}}[s_k[j] - 1, k] \vert , u_k[j]\} & \text { if } k \ge 2L. \end{array}\right. } \end{aligned}$$

It remains to compute the numbers $\vert {\mathcal {R}}[s_k[j] - 1, k] \vert$, for $j \in [1,|s_k|]$.

##### **Lemma 5**

*Consider a set of recombinants* ${\mathcal {R}} = \{R_1, \ldots , R_m\}$, *each of which has length* *n*. *For* $k \in [1,n]$ *and* $j \in [1,|s_k|]$, *one has* $\vert {\mathcal {R}}[s_k[j] - 1, k] \vert = t_k[j] + t_k[j + 1] + \cdots + t_k[|t_k|]$.

##### *Proof*

Denote $\ell = k - s_k[j] + 1$, so that ${\mathcal {R}}[s_k[j] - 1, k] = {\mathcal {R}}[k - \ell , k]$. Suppose that $\ell = 0$. Note that $R_{a_k[1]}[k] \le \cdots \le R_{a_k[m]}[k]$. Since $d_k[i] = k + 1$ iff either $i = 1$ or $R_{a_k[i-1]}[k] \ne R_{a_k[i]}[k]$, it is easy to see that $\vert {\mathcal {R}}[k,k] \vert$, the number of distinct letters $R_i[k]$, is equal to the number of time $k + 1 = s_k[|s_k|]$ occurs in $d_k$, i.e., $t_k[|t_k|]$.

Suppose that $\ell > 0$. It suffices to show that $\vert {\mathcal {R}}[k - \ell , k] \vert - \vert {\mathcal {R}}[k - \ell + 1, k] \vert = t_k[j]$. For $i \in [1,m]$, denote by $R'_i$ the string $R_i[k] R_i[k - 1] \cdots R_i[k - \ell ]$. Fix $w \in {\mathcal {R}}[k - \ell + 1, k]$. Since $R'_{a_k[1]} \le \cdots \le R'_{a_k[m]}$ lexicographically, there are numbers *h* and $h'$ such that $R_{a_k[i]}[k - \ell + 1, k] = w$ iff $i \in [h,h']$. Further, we have $R_{a_k[h]}[k - \ell ] \le R_{a_k[h + 1]}[k - \ell ] \le \cdots \le R_{a_k[h']}[k - \ell ]$. Thus, by definition of $d_k$, for $i \in [h + 1, h']$, we have $R_{a_k[i-1]}[k - \ell ] \ne R_{a_k[i]}[k - \ell ]$ iff $d_k[i] = k - \ell + 1 = s_k[j]$. Note that $d_k[h] > s_k[j]$. Therefore, the number of strings $R_i[k - \ell , k]$ from ${\mathcal {R}}[k - \ell , k]$ having suffix *w* is equal to one plus the number of integers $s_k[j]$ in the range $d_k[h, h']$, which implies $\vert {\mathcal {R}}[k - \ell , k] \vert - \vert {\mathcal {R}}[k - \ell + 1, k] \vert = t_k[j]$. $\square$

By (4) and Lemma 5, one can calculate *M*(*k*) in *O*(*m*) time using the arrays $t_k$ and $u_k$. 
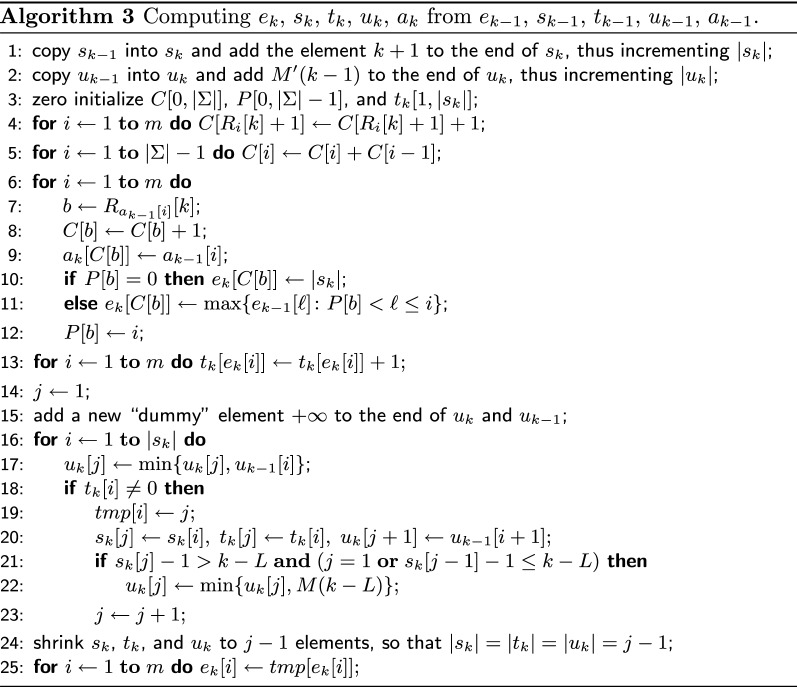


It remains to describe how we maintain $a_k, e_k, s_k, t_k, u_k$.

##### **Lemma 6**

*Algorithm* 3 *computes the arrays* $a_k, e_k, s_k, t_k, u_k$ *from the numbers* $M(k - L)$ *and* $M(k - 1)$, *and from the arrays* $a_{k-1}, e_{k-1}, s_{k-1}, t_{k-1}, u_{k-1}$ *in* *O*(*m*) *time, assuming the input alphabet is* $[0,|\Sigma |{-}1]$ *with* $|\Sigma | = O(m)$.

##### *Proof*

Let us analyze Algorithm 3 that computes $a_k, e_k, s_k, t_k, u_k$. By definition, $d_{k-1}[i] = s_{k-1}[e_{k-1}[i]]$ for $i \in [1,m]$. The first line of the algorithm initializes $s_k$ so that $d_{k-1}[i] = s_k[e_{k-1}[i]]$, for $i \in [1,m]$, and $s_k[|s_k|] = k + 1$. Since after this initialization $s_k$, obviously, is in the sorted order, one has, for $i, j \in [1,m]$, $e_{k-1}[i] \le e_{k-1}[j]$ iff $d_{k-1}[i] \le d_{k-1}[j]$ and, therefore, for $\ell \in [i,j]$, one has $d_{k-1}[\ell ] = \max \{d_{k-1}[\ell '] :i \le \ell ' \le j\}$ iff $e_{k-1}[\ell ] = \max \{e_{k-1}[\ell '] :i \le \ell ' \le j\}$. Based on this observation, we fill $e_k$ in lines 3–12 so that $d_k[i] = s_k[e_k[i]]$, for $i \in [1,m]$, using exactly the same approach as in Algorithm 1, where $d_k$ is computed, but instead of the assignment $d_k[C[b]] \leftarrow k + 1$, we have $e_k[C[b]] \leftarrow |s_k|$ since $s_k[|s_k|] = k + 1$. Here we also compute $a_k$ in the same way as in Algorithm 1.

The loop in line 13 fills $t_k$ so that, for $i \in [1,|s_k|]$, $t_k[i]$ is the number of occurrences of the integer *i* in $e_k$ ($t_k$ was zero initialized in line 3). Since, for $i \in [1,m]$, we have $d_k[i] = s_k[e_k[i]]$ at this point, $t_k[i]$ is also the number of occurrences of the integer $s_k[i]$ in $d_k[1,m]$.

By definition, $s_k$ must contain only elements from $d_k$, but this is not necessarily the case in line 14. In order to fix $s_k$ and $t_k$, we simply have to remove all elements $s_k[i]$ for which $t_k[i] = 0$, moving all remaining elements of $s_k$ and non-zero elements of $t_k$ to the left accordingly. Suppose that, for some *h* and *i*, we have $e_k[h] = i$ and the number $s_k[i]$ is moved to $s_k[j]$, for some $j < i$, as we fix $s_k$. Then, $e_k[h]$ must become *j*. We utilize an additional temporary array $tmp[1,|s_k|]$ to fix $e_k$. The loop in lines 16–23 fixes $s_k$ and $t_k$ in an obvious way; once $s_k[i]$ is moved to $s_k[j]$ during this process, we assign $tmp[i] = j$. Then, $s_k$, $t_k$, $u_k$ ($u_k$ is discussed below) are resized in line 24, and the loop in line 25 fixes $e_k$ using *tmp*.

Recall that $[s_k[j - 1] - 1, s_k[j] - 2]$, for $j \in [1,|s_k|]$, is a system of disjoint segments covering $[0, k - 1]$ (assuming $s_k[0] = 1$). It is now easy to see that this system is obtained from the system $[s_{k-1}[j - 1] - 1, s_{k-1}[j] - 2]$, with $j \in [1, |s_{k-1}|]$ (assuming $s_{k-1}[0] = 1$), by adding the new segment $[k - 1, k - 1]$ and joining some segments together. The second line of the algorithm copies $u_{k-1}$ into $u_k$ and adds $M'(k - 1)$ to the end of $u_k$, so that, for $j \in [1, |u_{k-1}|]$, $u_k[j]$ is equal to the minimum of $M'(\ell )$ for all $\ell$ from the segment $[s_{k-1}[j - 1] - 1, s_{k-1}[j] - 2]$ and $u_k[|u_{k-1}|{+}1] = M'(k - 1)$ is the minimum in the segment $[k - 1, k - 1]$. (This is not completely correct since $M'$ has changed as *k* was increased; namely, $M'(k - L)$ was equal to $+\infty$ but now is equal to $M(k - L)$). As we join segments removing some elements from $s_k$ in the loop 16–23, the array $u_k$ must be fixed accordingly: if $[s_k[j - 1] - 1, s_k[j] - 2]$ is obtained by joining $[s_{k-1}[h - 1] - 1, s_{k-1}[h] - 2]$, for $j' \le h \le j''$, then $u_k[j] = \min \{u_{k-1}[h] :j' \le h \le j''\}$. We perform such fixes in line 17, accumulating the latter minimum. We start accumulating a new minimum in line 20, assigning $u_k[j + 1] \leftarrow u_{k-1}[i + 1]$. If at this point the ready minimum accumulated in $u_k[j]$ corresponds to a segment containing the position $k - L$, we have to fix $u_k$ taking into account the new value $M'(k - L) = M(k - L)$; we do this in line 21. To avoid accessing out of range elements in $u_k$ and $u_{k-1}$ in line 20, we add a “dummy” element in, respectively, $u_k$ and $u_{k-1}$ in line 15. $\square$

Besides all the arrays of length *m*, Algorithm 3 also requires access to $M(k - L)$ and, possibly, to $M(k - 1)$. During the computation of *M*(*k*) for $k \in [1,n]$, we maintain the last *L* calculated numbers $M(k - 1), M(k - 2), \ldots , M(k - L)$ in a circular array, so that the overall required space is $O(m + L)$; when *k* is incremented, the array is modified in *O*(1) time in an obvious way. Thus, Lemma 6 implies Theorem 1

If, as in our case, one does not need $s_k, t_k, u_k$ for all *k*, the arrays $s_k$, $t_k$, $u_k$ can be modified in-place, i.e., $s_k$, $t_k$, $u_k$ can be considered as aliases for $s_{k-1}$, $t_{k-1}$, $u_{k-1}$, and yet the algorithm remains correct. Thus, we really need only 7 arrays in total: $a_k$, $a_{k-1}$, $e_k$, $e_{k-1}$, *s*, *t*, *u*, where *s*, *t*, *u* serve as $s_k$, $t_k$, $u_k$ and the array *tmp* can be organized in place of $a_{k-1}$ or $e_{k-1}$. It is easy to maintain along with each value $u_k[j]$ a corresponding position $\ell$ such that $u_k[j] = M'(\ell )$; these positions can be used then to restore the found segmentation of ${\mathcal {R}}$ using backtracking (see the beginning of the section). To compute $e_k$, instead of using an RMQ data structure, one can adapt in an obvious way Algorithm 2 rewriting the arrays $a_{k-1}$ and $e_{k-1}$ during the computation, which is faster in practice but theoretically takes $O(m\log |\Sigma |)$ time by Lemma 3. We do not discuss further details as they are straightforward.

#### From segmentation to founder set

Now we are given a segmentation ${\mathcal {S}}$ of ${\mathcal {R}}$ and we wish to produce a founder set ${\mathcal {F}}$ that obeys the segment boundaries. Recall that such founder set corresponds to a parse ${\mathcal {P}}$ of ${\mathcal {R}}$ with respect to segmentation ${\mathcal {S}}$. We conjecture that finding an optimal parse/founder set that minimizes the number of crossovers at segment boundaries is an NP-hard problem, but unfortunately we have not been able to prove this claim. Therefore, we continue by proposing three natural strategies of which two latter have interesting theoretical properties. The first of the strategies is a naive baseline, second is a greedy strategy, and third one is based on maximum weight perfect matching in a bipartite graph analogous to one by Ukkonen [18]. This latter strategy provides an optimal solution for a special case, and greedy gives a 2-approximation for the same special case. We will present all the three strategies first for the special case and then describe how to turn the general case to this special case (however loosing all optimality guarantees while doing so). We compare the naive baseline with the perfect matching in our experiments.

Assume (for our special case) that each segment in ${\mathcal {S}}$ induces exactly *M*(*n*) distinct substrings in ${\mathcal {R}}$. Then the naive baseline strategy to produce a founder set is to concatenate the distinct substrings of segment 1 with the distinct substrings of segment 2 in random order, and continue this process form left to right until *M*(*n*) founder sequences of length *n* are produced. For the latter two strategies, the idea is that instead of a random permutation, we aim to find a permutation that gives a concatenation order that minimizes the number of crossovers at each segment boundary. For this purpose, it is sufficient to consider two consecutive segments [*a*, *b*] and $[b+1,c]$ as two *partitions* of the rows of ${\mathcal {R}}$. Namely, consider a distinct substring *X* of a segment [*a*, *b*] and an induced set $A \subseteq \{1,2,\ldots m\}$ such that $R_i[a,b]=X$ for all $i\in A$. Analogously, consider a distinct substring *Y* of a segment $[b+1,c]$ and an induced set $B \subseteq \{1,2,\ldots m\}$ such that $R_i[b+1,c]=Y$ for all $i\in B$. If the concatenation *XY* forms the content *F*[*a*, *c*] of some founder *F*, then this concatenation causes $m-\vert A\cap B \vert$ crossovers. Hence, to minimize crossovers, one seeks to maximize the intersection between two partitions, studied next.

*Problem of maximum intersection between two partitions.* Let *a* be an integer. Given two partitions $E_1$ and $E_2$ of $\{1,\ldots ,a\}$ with $\vert E_1 \vert = \vert E_2 \vert$, the problem of *Maximum Intersection Between two Partitions* (MIBP) is to find the bijection *f* from $E_1$ to $E_2$ which maximizes $\sum _{x \in E_1} \vert x \cap f(x) \vert$.

By using the bipartite graph defined between the elements of $E_1$ and the elements of $E_2$ and such that for $x \in E_1$ and $y \in E_2$, the weight of this edge is $w(x,y) = \vert x \cap y \vert$, a maximum weight perfect matching of this graph gives an optimal solution of MIBP, and hence this problem can be solved in polynomial time.

We can define the greedy algorithm related to MIBP as the the greedy algorithm related to the problem of maximum weight perfect matching in the previous bipartite graph. As the greedy algorithm for maximum weight perfect matching is $\frac{1}{2}$-approximation [24], we have the same ratio of approximation for the greedy algorithm for MIBP.

##### **Lemma 7**

*Let* $E_1$ *and* $E_2$ *be two partitions of* $\{1,\ldots ,a\}$ *with* $\vert E_1 \vert = \vert E_2 \vert$. *We can compute the greedy algorithm for MIBP of* $E_1$ *and* $E_2$ *in* *O*(*a*) *time.*

##### *Proof*

Let *E* be a partition of $\{1,\ldots ,a\}$ and $\prec$ be a total order on *E*, we denote by $G_E$ the array of elements of *E* of size *a* such that for all *i*, $G_E[i] = e_i$ where $i \in e_i \in E$. Let be $x \in E_1$ and $y \in E_2$. We have $w(x,y) = \vert x \cap y \vert = \vert \{i \in \{1,\ldots ,a\} \ | \ i \in x \cap y\} \vert = \vert \{i \in \{1,\ldots ,a\} \ | \ G_{E_1}[i] = x \text { and } G_{E_2}[i] = y \} \vert$. It follows that the number of edges of no zero weight is at most *a*. By using Radix sort, we can compute in *O*(*a*) the sorted array of elements of $\{1,\ldots ,a\}$ following the order where $i<j$ iff $G_{E_1}[i] \prec G_{E_1}[j]$ or $G_{E_1}[i] = G_{E_1}[j]$ and $G_{E_2}[i] \prec G_{E_2}[j]$. With this array, as for all $x \in E_1$ and $y \in E_2$ $w(x,y) \le a$, we can compute (by further Radix sort and renaming steps) in *O*(*a*) the ordered list $[(x_1,y_1),\ldots ,(x_q,y_q)]$ such that $w(x_1,y_1) \ge \cdots \ge w(x_q,y_q) > 0$ with $q \le a$. By taking the elements in the order of this list, we can compute in *O*(*a*) two arrays *f* and $f^{-1}$ of size $\vert E_1 \vert$ such that $\{(i,f[i]) \ | \ i \in E_1 \}$ and $\{(f^{-1}[i],i) \ | \ i \in E_2 \}$ represent the same solution of the greedy algorithm for MIBP. $\square$

*Optimal founder set for the special case.* Now we can solve independently the MIBP problem for each pair of consecutive segments, resulting to the following theorems, where the first one follows directly also from earlier constructions [18], and the latter from Lemma 7.

##### **Theorem 8**

([18]) *Given a segmentation* ${\mathcal {S}}$ *of* ${\mathcal {R}}$ *such that each segment induces exactly* *K* *distinct substrings in* ${\mathcal {R}}$, *then we can construct an optimal parse* ${\mathcal {P}}$ *of* ${\mathcal {R}}$ *(and hence the corresponding set of founders) in polynomial time.*

##### **Theorem 9**

*Given a segmentation* ${\mathcal {S}}$ *of* ${\mathcal {R}}$ *such that each segment induces exactly* *K* *distinct substrings in* ${\mathcal {R}}$, *then we can construct a greedy parse* ${\mathcal {P}}$ *of* ${\mathcal {R}}$ *(and hence the corresponding set of founders) that has at most twice as many crossovers than the optimal parse in* $O(\vert {\mathcal {S}} \vert \times m )$ *time and* $O(\vert {\mathcal {S}} \vert \times m )$ *space.*

**Fig. 3:**
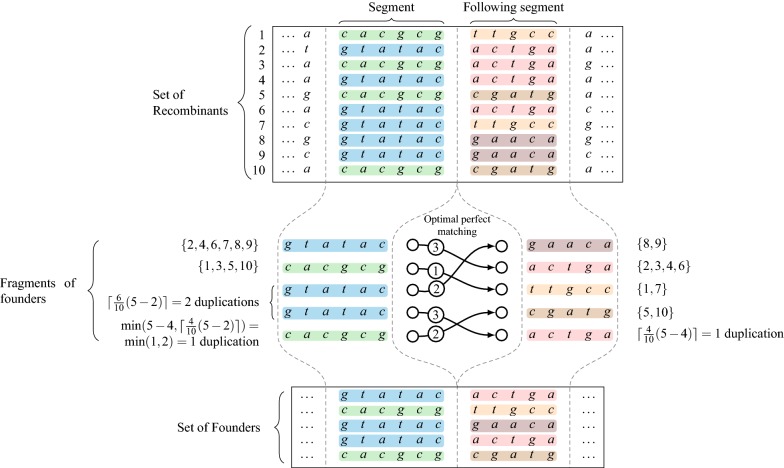
The duplication of the fragments and the link between optimal solution of perfect matching and the concatenation of the fragments to obtain the set of founder sequences

In the general case, there are segments inducing less than *M*(*n*) distinct substrings. We turn such segments to the special case by duplicating some of the substrings. The choices made have dependencies between segments, and this is the reason we believe this general case is NP-hard to solve optimally. Hence, we aim just to locally optimize the chances of minimizing crossovers by duplicating distinct substrings in proportion they cover ${\mathcal {R}}$. That is, consider a segment inducing $k<M(n)$ distinct substrings and the corresponding partitioning *E* of $\{1,\ldots ,m\}$. Consider the largest set *x* of *E*. We make $k_x=\lceil \frac{\vert x \vert }{m}(M(n)-k)\rceil$ copies of the corresponding distinct substring. We continue by decreasing cardinality, stop when the sum of the duplication counts is greater than or equal to *M*(*n*) and update the last one such that $k + \Sigma _i k_i = M(n)$ (see Fig. 3). The fact that the corresponding partitioning is now a multi-partitioning (containing same set multiple times), does not affect the functioning of the greedy or perfect matching algorithms for the MIBP problem.

## Results

We implemented the segmentation algorithm using Algorithm 2 to build the pBWT arrays and computed the minimum number of founders with the given value of *L* using the recursion in Eq. 3. This part of the implementation corresponds to Lemma 3, and thus the overall time complexity of the implemented approach is $O(mn \log |\Sigma |)$. After computing the minimum number of founders, we use backtracking to determine the optimal segmentation. Since we use the pBWT arrays to determine the distinct substrings in each segment, as part of the first phase of building the arrays we also store samples and now update them to the segment boundary positions in parallel. We proceed to join adjacent segments from left to right until the number of distinct substrings in one segment would exceed the minimum number of founders, and finally we concatenate the substrings to generate founder sequences. The implementation outputs for each segment the distinct founder sequence fragments, and associates to each fragment the set of haplotypes containing that fragment as a substring at that location (these are easily deduced given the segmentation and the positional BWT structures). Our implementation uses integer vectors from the SDSL library [25].

As our goal is to produce reference sequences for aligning short reads, we wanted to find a good value of *L* to generate a segmentation suitable for this purpose. In particular, we wanted to have the length of most segments clearly above a typical read length, such that most reads could be aligned without hitting a recombination site.

We used the chromosome 6 variants from the phase 3 data of the 1000 Genomes Project [2] as the starting point. We converted the variant data to a multiple sequence alignment with vcf2multialign,^1^ which resulted in 5009 haplotype sequences of equal length (including the reference sequence) of approximately 171 million characters. In order to reduce the running time of our tool, we discarded columns of identical characters as they would not affect the number of recombination sites. This reduced each sequence to approximately 5.38 million characters.

**Fig. 4:**
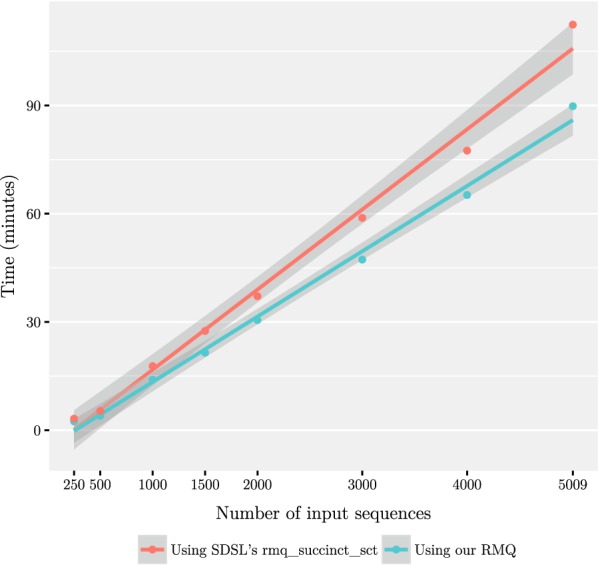
The running time of our implementation plotted against the number of input sequences with $L = 10$ and using either our RMQ data structure or rmq_succinct_sct from SDSL. The data points have been fitted with a least-squares linear model, and the grey band shows the 95% confidence interval

We used an increasing number of the generated sequences as an input to our tool with the value of *L* fixed to 10 to verify the usability of the tool in terms of running time and memory consumption. The tests were run on a Ubuntu Linux 16.04 server. The server had 96 Intel Xeon E7-4830 v3 CPUs running at 2.10GHz and 1.4 TB of memory. In addition to our own RMQ data structure, we tested with a general-purpose RMQ from the SDSL library. As seen in Fig. 4, our special-purpose RMQ data structure performed somewhat better in terms of speed compared to the general-purpose library implementation. From this experiment it is conceivable that processing of thousands of complete human genomes takes only few CPU days. As we did not optimize the memory usage of our tool, the maximum resident set size with 5009 inputs was around 257 GB which corresponds to approximately 10.25 bytes per input character. We expect that the memory consumption may be reduced without much affecting the performance.

**Fig. 5:**
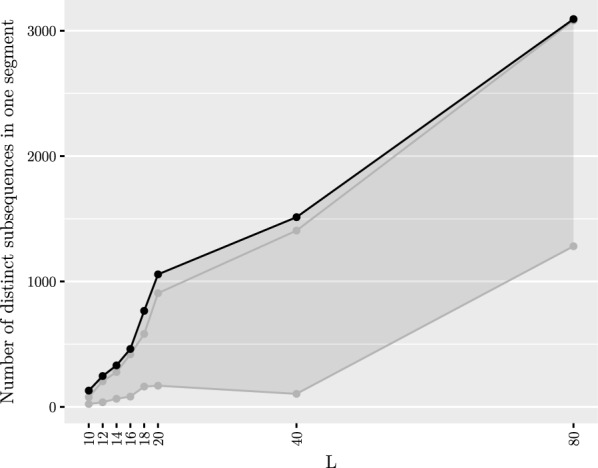
Maximum (shown in black)/median/minimum number of distinct subsequences in one segment given a set of founder sequences generated with a set of 5009 input sequences

**Fig. 6:**
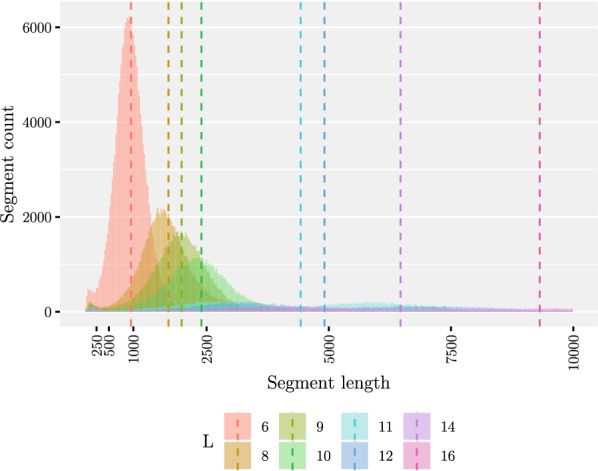
Distribution of segment lengths in the range [0, 10000) given a set of founder sequences generated from a set of 5009 input sequences and varying the value of *L*. Only the resulting segmentations with the values $L \in \{6,8,9,10, 11,12, 14, 16\}$ have been plotted since the other ones were not visible. The mean values are shown with the dashed lines

**Fig. 7:**
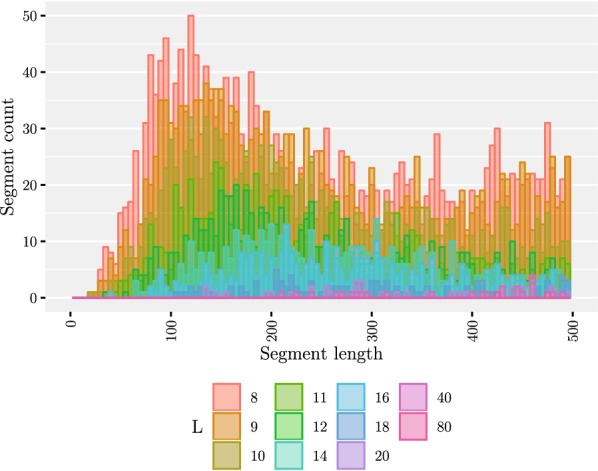
Distribution of segment lengths in the range [0, 500) given a set of founder sequences generated from a set of 5009 input sequences and varying the value of *L*

Our second experiment was to see the effect of the minimum length *L* on the number of founders as well as the length of the segments. The results have been summarized in Table 1. We tested with a number of values of *L* ranging from 10 to 80. After generating the founders, we mapped the segment co-ordinates back to the original sequences to determine the segment lengths. The results are shown in Figs. 5 and 6. We note that while the average segment length of 2395 bases with $L = 10$ is fitting our purpose, there is a peak of short segments of approximately 250 bases. The peak is magnified in Fig. 7. We also tested smaller values of *L* to conclude that decreasing *L* further rapidly makes the situation more difficult. On the other hand, setting $L = 10$ resulted in only 130 founders, which makes aligning reads much faster than using all of the haplotypes for indexing.

**Table 1:** Summarized results with 5009 input sequences

L	Number of founders	Average segment length	Median number of recombinations	Average distance between recombinations
10	130	2395	15,794	9624
12	246	4910	11,716	14,025
14	331	6467	9759	17,126
16	462	9312	7801	21,860
18	766	14,383	5593	30,571
20	1057	20,151	4411	39,090
40	1513	30,228	3228	54,386
80	3093	67,994	1176	146,655

We measured the average segment length from the segmentation, median number of recombinations from mapping the input sequences to the founder sequences, and average distance between recombinations by dividing the length of the original sequences by the average number of recombinations. The last three columns report the results for the perfect matching approach

**Fig. 8:**
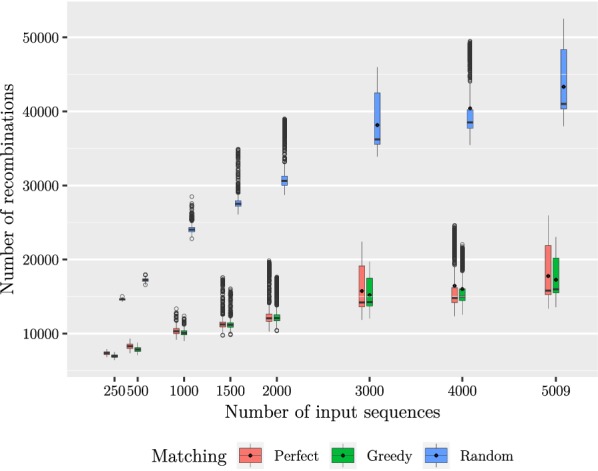
Number of recombinations in one input sequence given a set of founder sequences generated with a varying number of input sequences and $L = 10$. Here the median is displayed inside each box, the lower and upper hinges correspond to the first and third quartiles, and the data points outside the range of 1.5 times the distance between the first and the third quartiles from the hinges have been plotted individually. The mean values are shown with black diamonds for 3000, 4000 and 5009 input sequences. The experiments were done with the eight inputs listed on the x axis. The plotted boxes have been shifted slightly in order to prevent overprinting

**Fig. 9:**
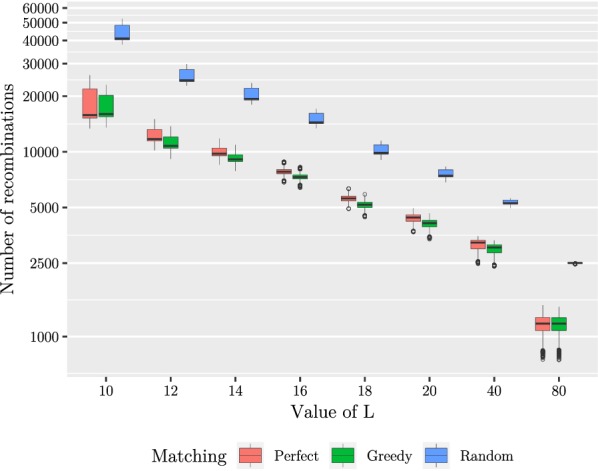
Number of recombinations in one input sequence given a set of founder sequences generated from a set of 5009 input sequences and varying the value of *L*. See Fig. 8 for description of visualization details

We proceeded with two tests in which we measured the number of recombinations needed to express each of the original sequences with the generated founder sequences depending on the method of concatenating the fragments into the set of founder sequences. Using the method given earlier, we began by duplicating some fragments so that each segment had exactly the same amount of fragments. For these tests, we implemented the three concatenation strategies: a *Random matching* which corresponds to concatenating the consecutive fragments in random order, a *Perfect matching* which takes an optimal solution of the maximum weight perfect matching problem as the order for the concatenation of the fragments, and a *Greedy matching* which solves the matching problem greedily. For evaluating the different concatenation strategies, we mapped each one of the original sequences to the founders, using a simple greedy algorithm that is also optimal [19]. In the first test, we fixed the value of *L* to 10 and mapped an increasing number of input sequences to a set of founder sequences generated with the same input sequences. In the second one, we used all of the 5009 input sequences and varied the value of *L*. The results are shown in Figs. 8 and 9. Considering the 17768 and 43333 recombinations achieved with perfect and random matching, respectively, given 5009 input sequences and $L = 10$ (see Table 1), we conclude that the heuristic part of optimizing the concatenation of founder blocks yields an improvement of around 2.44 compared to a random concatenation of segments with duplications. Greedy approach works even slighly better than perfect matching in our experiments: the number of recombinations on the same setting is 17268. As the numbers are very close, we refer to perfect matching numbers in the sequel.

The results look promising, as using 130 founders instead of 5009 haplotypes as the input to our pan-genome indexing approach [12] will result into significant saving of resources; this solves the space bottleneck, and the preprocessing of founder reconstruction also saves time in the heavy indexing steps.

Our intention was to compare our tool to an implementation of Ukkonen’s algorithm [19]. However, initial testing with four input sequences showed that the latter implementation is not practical with a data set of this size.

## Conclusions

As our experiments indicate that one can reduce 5009 haplotypes down to 130 founders with the average distance of two crossovers being 9624 bases, one can expect short read alignment and variant calling to become practical on such pan-genomic setting. We are investigating this on our tool *PanVC* [12], where one can simply replace its input multiple alignment with the one made of the founder sequences. With graph-based approaches, slightly more effort is required: Input variations are encoded with respect to the reference, so one first needs to convert variants into a multiple alignment, apply the founder reconstruction algorithm, and finally convert the multiple alignment of founder sequences into a directed acyclic graph. PanVC toolbox provides the required conversions. Alternatively, one can construct the pan-genome graph using other methods, and map the founder sequences afterwards to the paths of the graph: If original haplotype sequences are already spelled as paths, each founder sequence is a concatenation of existing subpaths, and can hence be mapped to a continuous path without alignment (possibly requiring adding a few missing edges).

Finally, it will be interesting to see how much the contiguity of the founder sequences can still be improved with different formulations of the segmentation problem. We are investigating a variant with the number of founder sequenced fixed.

## Data Availability

Our implementation is open source and available at the URL https://github.com/tsnorri/founder-sequences.

## Abbreviations

pBWT: positional Burrows–Wheeler transform
LCP: longest common prefix
RMQ: range maximum query
MIBP: maximum intersection between two partitions

## Footnotes

1 https://github.com/tsnorri/vcf2multialign.
